# An Introduction to Practical Chemistry, Including Analyses

**Published:** 1867-02

**Authors:** 


*An Introduction to Practical Chemistry, including Analyses.* By John E. Bowman, F. C. S., late Professor of Practical Chemistry in King's College, London. Edited by Charles Bloxam, F. C. S., Professor of Practical Chemistry in King's College, London, &c., with one thousand and seven illustrations. Fourth American, from the fifth London edition revised.

We have heard this work well spoken of.

